# Incidence of Weaning Failure in Obese Patients in Intensive Care Unit

**DOI:** 10.7759/cureus.55881

**Published:** 2024-03-10

**Authors:** Anum Ilyas, Nusrat Kharadi, Mudassir Shafique, Tooba Mehreen, Maria Habib, Jaffar Khan, Aftab Akhtar, FNU Kiran, Farrukh Mehmood

**Affiliations:** 1 Critical Care Department, Shifa International Hospital Islamabad, Islamabad, PAK; 2 Pulmonology Department, Islamabad Medical Complex, Islamabad, PAK; 3 Internal Medicine Department, Shifa International Hospital Islamabad, Islamabad, PAK; 4 Internal Medicine Department, Liaquat National Hospital and Medical College, Karachi, PAK

**Keywords:** ventilator weaning, positive end-expiratory pressure (peep), peep, morbidly obese, mechanical ventilation

## Abstract

Background

The escalating prevalence of obesity worldwide presents unique challenges in critical care management, especially in the context of mechanical ventilation and weaning processes in intensive care units (ICUs). The present study aimed to determine the incidence of weaning failure in obese patients in an ICU.

Methods

A prospective observational study was carried out to gather data on patients in the ICU of Shifa International Hospital located in Islamabad, Pakistan. The target population consisted of adult patients who were both male and female, ages 18 years and above. These individuals required intubation procedures as well as mechanical ventilation during their hospitalization. The researchers followed these patients prospectively and observed their medical conditions closely to gather data about how obesity might impact critical care interventions and outcomes.

Results

The sample size was 288 bearing a median age of 61.0 with an interquartile range of 19 years. Older age manifested a significantly higher frequency of failed extubation (p=0.065). Higher body mass index (BMI) was significantly associated with failed extubation among the study population. It was found that a higher significant difference was associated with BMI > 30 kg/m^2^ (obese) in failed and successful extubation. One-half of the patients with failed extubation and only 16 (5.9%) patients with successful extubation had end-stage renal disease (p<0.001). It was found that patients who underwent failed extubation had notably increased ICU mortality (p=0.108), 28-day mortality (p=0.067), as well as mean ICU (p<0.001) and hospital stay (p=0.007).

Conclusion

Our study revealed some insightful correlations between obesity, age, comorbidities, length of hospitalization, ICU stay, and mortality rate in terms of weaning failure among the study population.

## Introduction

The World Health Organization (WHO) describes obesity as a multifactorial chronic condition marked by an abnormally raised amount of adipose tissues in the body. Obesity is a growing global issue. Statistically, 42% of the Western population is classified as obese (body mass index (BMI) > 30), and 9.2% fall under obese class III (BMI > 40) [1]. The findings show a comparable trend in the East [2]. Obesity is strongly correlated to an elevated risk of hypercapnic respiratory failure, increased mechanical ventilation period, and longer weaning intervals [3,4]. Obese patients invariably end up in the ICU, where invasive mechanical ventilation (IMV) stands as a frequently employed treatment alternative, owing to the increasing prevalence of this metabolic syndrome [5]. This subset of critical patients poses unique challenges because of their greater morbidity rates, irrespective of the reason for hospitalization [6-8]. Ample amount of studies investigated the connection amid obesity and ICU mortality yielding contrasting results [6,7].

A study by Brandenberger et al. [8] determined that 34% of the mechanically ventilated subjects possessed a normal BMI. Both underweight and obese patients exhibited a longer ventilation duration as compared to normal weight patients [8]. Underweight and morbidly obese subset of patients tend to require additional care and intervention during their ICU stays to address the concomitant issues of an abnormal BMI.

Failure to wean from ventilator among obese patients is attributable to the inadequacy of the traditionally defined positive end-expiratory pressure (PEEP) levels to induce appropriate airway pressures required to counter the opposing pleural pressures and reduced chest wall compliance that results from abnormal fat buildup in the torso [9-15]. When weaning obese people, PEEP modifications using an esophageal balloon to retain a positive pressure time product (Ptp) between 0 and 5 cm H_2_O (aiming to maintain it near to 0 as feasible) or relevant adjustments to obtain the best static compliance (Cstat) may generate better outcomes [12].

The optimization of ventilatory support constitutes one of the most complicated factors in treating severely ill obese individuals. This study is aimed at assessing the incidence of weaning failure in obese individuals and exploring potential mechanical ventilation techniques to restore the new optimal lung capacity.

## Materials and methods

A prospective observational study was carried out to gather data on patients in the intensive care unit of Shifa International Hospital located in Islamabad, Pakistan. We sought ethical approval from the institutional review board prior to data collection. The recruitment process was implemented through the use of non-probability consecutive sampling techniques, which ensured that the participants were selected systematically and without any bias. These methods allowed for an accurate representation of the patient population at the hospital and ensured that all necessary precautions were taken to maintain ethical standards during data collection.

Sample size 288 was determined by utilizing the WHO sample size calculator based on a reference proportion of weaning failure of 25%, as reported in a study by Obi et al. [3]. The calculation also incorporated a margin of error of 5% and a confidence level of 95%. Prior to the trial, all participants and their legal guardians were requested to provide informed verbal and written consent. The study focused on a group of individuals who were dealing with obesity and required medical assistance in the intensive care unit. The participants selected for this research were adults, both males and females, whose body mass index (BMI) was equal to or higher than 30, considered an alarming level of obesity. Moreover, these participants were over 18 years old and had to undergo intubation and mechanical ventilation services to aid them in their treatment. The researchers observed and analyzed various aspects related to the patients' health conditions during their stay in the ICU.

The study excluded patients with any structural abnormalities that hindered ventilatory support. Patients with pulmonary, neuromuscular, or cardiac diseases that could prevent them from discontinuing ventilator support were also excluded from the study. The study began after procuring the clearance from the College of Physician and Surgeons of Pakistan (CPSP) and approval of the ethical review committee of the department.

The assessment of obesity was conducted among all individuals admitted to the intensive care unit. The categorization of patients was based on their BMI status. Patients with a BMI ranging from 18 to 24 were categorized as having a healthy weight, those with a BMI below 18 were considered underweight, and those within the range of 24.9-29.9 were classified as overweight. At the same time, those whose BMI measured at 30 kg/m^2^ or above were identified as obese. Weight was determined in kilograms and height in meters using appropriate measuring scales to obtain the BMI measurements.

During the research period, comprehensive documentation was conducted containing the medical records of all patients admitted to the medical intensive care unit at Shifa International Hospital and remained there for more than 21 days. The primary outcome of the study was the outcome of weaning, and patients will be divided according to the outcomes, i.e., I) successful weaning, and II) failed weaning. All patients who have their first successful trial with spontaneous breathing were defined as “successful weaning”. A predefined pro forma was used to collect data from the patients.

Data analysis was conducted using the 24th Statistical Package for Social Sciences (SPSS) version. All data including duration of mechanical ventilation, comorbid conditions like hypertension, diabetes mellitus, chronic obstructive pulmonary disease (COPD), and other clinically significant variables were noted. In order to present the findings, medians or interquartile ranges were used for continuous variables such as age and hospitalization and then compared with the Mann-Whitney U tests. The categorical variables, which encompassed gender, outcome of weaning, and comorbidities, were presented either as a number or percentage and then compared using chi-squared tests. An assessment was conducted to examine the relationship between each variable and weaning success as well as ICU and in-hospital mortality rates. A chi-square test was utilized in conducting a post-stratification analysis to ascertain the effects of gender, age, comorbidities, COPD, and obstructive sleep apnea (OSA) on the outcome of weaning. The statistical significance threshold was established at a p-value of ≤ 0.05.

## Results

The study included 288 individuals with a median age of 61 with an interquartile range (IQR) of 19 years. The majority of the subjects were male and had a median body mass index (BMI) of 31.7. The median length of hospital stays was 28 days, and their median duration of stay in the ICU was nine days (Table 1).

**Table 1:** Patient sociodemographic and clinical history ICU: intensive care unit, IQR: interquartile range.

Characteristics	Mean/N	Median (IQR)
Age (years)	62.5 ± 13.2	61.0 (19)
Gender		
Male	195 (67.7%)	
Female	93 (32.3%)	
Body mass index	32.0 ± 1.2	31.7 (1.0)
Duration of hospital stay	27.6 ± 10.61	28.0 (12)
Duration of ICU stay	10.5 ± 5.2	9.0 (9)
Respiratory failure	70 (24.3%)	
Etiology of respiratory failure		
Pulmonary system	70 (24.3%)	
Neurological system	68 (23.6%)	
Cardiovascular system	64 (22.2%)	
Gastrointestinal system	43 (14.9%)	
Comorbidities		
Diabetes mellitus type 2	110 (38.2%)	
Hypertension	105 (36.5%)	
Chronic obstructive pulmonary disease (COPD)	29 (10.1%)	
Ischemic heart disease	50 (17.4%)	

Higher BMI was significantly associated with failed extubation among the study population. Sixteen patients had failed extubation, while 272 had successful extubation. Cardiovascular disease was associated with a significantly higher frequency of failed extubation (p=0.033). One-half of the patients with failed extubation and only 16 (5.9%) patients with successful extubation had end-stage renal disease (p< 0.001) (Table 2).

**Table 2:** Comparison of failed and successful extubation groups APACHE: Acute physiology and chronic health evaluation, TISS: Therapeutic Intervention Scoring System, COPD: chronic obstructive pulmonary disease.

Variable	Failed extubation	Successful extubation	p-value
Age (years)	68.5 ± 14.7	62.2 ± 13.1	0.065
Male	9 (56.3%)	186 (68.4%)	0.313
Body mass index (BMI) (kg/m^2^)	33.5 ± 3.1	31.9 ± 0.95	<0.001
Mean APACHE II	19.2 ± 4.5	17.1 ± 3.2	0.014
TISS Scale	32.7 ± 7.8	27.9 ± 4.7	<0.001
Glasgow coma scale	11.4 ± 3.2	11.7 ± 2.5	0.691
Number of comorbidities	1.8 ± 0.6	1.1 ± 0.3	<0.001
Comorbidity			
End-stage renal disease	8 (50%)	16 (5.9%)	<0.001
Diabetes mellitus (DM)	7 (43.8%)	103 (37.9%)	0.638
Stroke	6 (37.5%)	68 (25%)	0.266
Coronary artery disease (CAD)	4 (25%)	80 (29.4%)	0.477
Cancer	2 (12.5%)	37 (13.6%)	0.629
COPD	1 (6.3%)	28 (10.3%)	0.506
Liver cirrhosis	0 (0%)	9 (3.3%)	0.593
Cause of respiratory failure			
Pulmonary system	3 (18.8%)	67 (24.6%)	0.426
Cardiovascular system	7 (43.8%)	57 (21%)	0.033
Neurological system	4 (25%)	64 (23.5%)	0.547
Renal system	1 (6.3%)	17 (6.3%)	0.737
Gastrointestinal system	1 (6.3%)	42 (15.4%)	0.278
Others	0 (0%)	23 (8.5%)	0.254

Table 3 presents a comparison of the results between failed and successful extubation. The study indicated that patients who underwent failed extubation had notably increased ICU mortality (p=0.108), 28-day mortality (p=0.067), as well as mean ICU (p<0.001) and hospital stay (0.007).

**Table 3:** Outcomes of failed and successful extubation groups ICU: intensive care unit.

Parameter	Total	Failed extubation	Successful extubation	p-value
ICU mortality	2 (0.7%)	1 (6.3%)	1 (0.4%)	0.108
28-day mortality	8 (2.8%)	2 (12.5%)	6 (2.2%)	0.067
ICU stay (days)	10.5 ± 5.2	15.4 ± 6.9	10.2 ± 5.0	<0.001
Hospital stay (days)	27.6 ± 10.6	34.5 ± 12.9	27.2 ± 10.3	0.007

## Discussion

Obesity is highly correlated with an increased mortality rate in the general population [13-15]. However, its association with ventilatory support modalities and its mortality in the ICU has been controversial. Likened to the current findings, the meta-analysis by Zhao et al. demonstrated that obese patients associated with mechanical ventilation in the ICU had relatively reduced ICU mortality (odds ratio (OR)=0.88, 95% CI: 0.0.84-0.92, I^2^=0%), hospital mortality (OR=0.83, 95% CI: 0.74-0.93, I^2^=52%), short-term mortality (OR=0.81, 95% CI: 0.74-0.88, I^2^=0%), as well as long-term mortality (OR=0.69, 95% CI: 0.60-0.79, I^2^=0%) as compared to the subset of patients with normal BMI, irrespective of length of the ventilation period [14].

Various studies have shown that obese patients necessitate mechanical ventilation for an extended period of time than non-obese patients, with a mean ventilation period of 2.17 to 15.2 days and 1.86 to 13.2 days, respectively [14-17]. This is due to higher vulnerability to atelectasis or heightened alveolar tension due to poor lung and chest wall compliance [17]. With the extubation failures, the length of hospitalization consequently increases with the need for reintubation as depicted by our patient outcomes. However, the success of extubation did not show any significant dependency on the BMI of the patients, i.e., the p-value was 0.433 in both overweight and obese classes. Our ICU mortality in failed extubation turned out to be 6.3% which stands markedly higher than the 0.4% of successful extubations, reinforced by the near-significant difference in 28-day mortality among the failed and successful extubations. In line with these results, by distinguishing between extubation failure and weaning failure, one may see the need for pre-extubation testing that concentrates on determining patent airway in addition to assessing the restored lung function. Tracheal reintubation following planned extubation is reported to occur between 0.1% and 0.45% of the time following elective procedures in operating rooms and postanesthesia care units, as opposed to more frequent incidences in critically sick patients, i.e., 0.4%-25% with a higher risk of mortality [18].

Obesity, obstetrics, cervical spine surgeries, significant head/neck and upper airway surgery, obstructive sleep apnea, and other conditions bear much higher chances of extubation failure and are commonly associated with challenging airway monitoring. There is a lack of upper airway patency after extubation failure owing to edema, and soft tissue collapse, followed by laryngospasm [18,19]. This incurs the need for a comprehensive airway care approach including planning for tracheal extubation, especially when working with patients whose airways are complex or in conditions where the risk of extubation failure is higher such as in obesity. It is essential to properly prepare by identifying patients with challenging airways or at risk of developing them, understanding situations that may increase the likelihood of post-extubation airway complications, and evaluating the fundamental reasons and underlying mechanisms for extubation incompetence. An effective strategy to mitigate post-extubation airway complications should include preemptive optimization of patients' conditions, precise extubation timing, well-informed staff proficient in advanced airway management, accessibility to the necessary equipment, and appropriate monitoring after extubation [18,20].

Another study by Obi et al. discusses the role of the esophageal balloon (ESO) and static compliance (Cstat) in the weaning states of obese patients admitted to the ICU. Upon evaluation, the results suggested that a month later, no significant variation was noted in the percentage of individuals weaned, with 62% and 75% in the ESO group and the Cstat groups, respectively (p=0.67). Among the patients who weaned, employment of the ESO exhibited a swifter and significantly quicker liberation from mechanical ventilation (3.5 days of ESO group vs 14 days of Cstat group; p=0.01). The mean of optimal PEEP in the ESO and Cstat groups was similar; 26.5 ± 5.7 cmH_2_O and Cstat 24.2 ± 7 cmH_2_O (p=0.38), respectively [1]. Currently, the outcome is devoid of any rational explanation; however, it is highly anticipated that there might be more fundamental institutional elements at play.

The limitations of this study come from its small sample size, the non-probability consecutive sampling technique in the selection of participants that may stem a bias, and its cross-sectional format. Although speculative, the "obesity paradox" continues to be a crucial subject in terms of mechanical ventilation in intensive care units. This area demands rigorous research to determine the possible correlation of obesity with the risk of hospital mortality.

## Conclusions

Our study revealed some insightful correlations between obesity, age, comorbidities, hospital and ICU stay, and mortality with failure of weaning among the study population. In future studies, we plan to do a more comprehensive evaluation in multiple hospitals to gather a larger sample. It is crucial to demonstrate causal connections and predictors of weaning failure. The development of predictive models, prospective studies, and randomized controlled trials should be the main objectives of future studies. This will make it easier to wean patients off of artificial ventilation, speed up the process, allow for early interventions, and give a comprehensive picture of the difficulties involved.
